# Agricultural Pesticide Use and Hypospadias in Eastern Arkansas

**DOI:** 10.1289/ehp.9146

**Published:** 2006-07-06

**Authors:** Kristy J. Meyer, John S. Reif, D.N. Rao Veeramachaneni, Thomas J. Luben, Bridget S. Mosley, John R. Nuckols

**Affiliations:** 1 Department of Environmental and Radiological Health Sciences, Colorado State University, Fort Collins, Colorado, USA; 2 Department of Biomedical Sciences, Colorado State University, Fort Collins, Colorado, USA; 3 Arkansas Center for Birth Defects Research and Prevention, University of Arkansas for Medical Sciences and Arkansas Children’s Hospital Research Institute, Little Rock, Arkansas, USA

**Keywords:** endocrine disruption, exposure assessment, hypospadias, male urogenital disorders, pesticides

## Abstract

**Introduction:**

We assessed the relationship between hypospadias and proximity to agricultural pesticide applications using a GIS-based exposure method.

**Methods:**

We obtained information for 354 cases of hypospadias born between 1998 and 2002 in eastern Arkansas; 727 controls were selected from birth certificates. We classified exposure on pounds of pesticides (estimated by crop type) applied or persisting within 500 m of each subject’s home during gestational weeks 6 to 16. We restricted our analyses to 38 pesticides with some evidence of reproductive, developmental, estrogenic, and/or antiandrogenic effects. We estimated timing of pesticide applications using crop phenology and published records.

**Results:**

Gestational age at birth [odds ratio (OR) = 0.91; 95% confidence interval (CI), 0.83–0.99], parity (OR = 0.79; 95% CI, 0.65–0.95), and delaying prenatal care until the third trimester (OR = 4.04; 95% CI, 1.46–11.23) were significantly associated with hypospadias. Risk of hypospadias increased by 8% for every 0.05-pound increase in estimated exposure to diclofop-methyl use (OR = 1.08; 95% CI, 1.01–1.15). Pesticide applications in aggregate (OR = 0.82; 95% CI, 0.70–0.96) and applications of alachlor (OR = 0.56; 95% CI, 0.35–0.89) and permethrin (OR = 0.37; 95% CI, 0.16–0.86) were negatively associated with hypospadias.

**Conclusions:**

Except for diclofop-methyl, we did not find evidence that estimated exposure to pesticides known to have reproductive, developmental, or endocrine-disrupting effects increases risk of hypospadias. Further research on the potential effects of exposure to diclofop-methyl is recommended.

Several studies suggest that the incidence of developmental and acquired abnormalities of the male urogenital system, including testicular cancer and cryptorchidism, has increased since 1970 (Giwercman et al. 1993; Paulozzi 1999). A concurrent worldwide increase in the production and use of synthetic chemicals including pesticides suggests that environmental factors may be at least partly responsible (Toppari et al. 1996). The evidence that incidence of specific birth defects such as hypospadias is increasing is less clear; hypospadias incidence was found to be unchanged in California between 1984 and 1997 (Carmichael et al. 2003). Androgen levels are critical in the development of male external genitalia (Baskin 2004). Estrogenic and antiandrogenic substances can disturb normal urogenital development by disrupting hormone dependent pathways (Steinhardt 2004). This can lead to impaired Sertoli and/or Leydig cell function, resulting in manifestations such as hypospadias, cryptorchidism, and testicular cancer, collectively termed testicular dysgenesis syndrome (Skakkebæk et al. 2001).

Hypospadias is a congenital malformation of the urethra with a multifactorial etiology. In children with hypospadias, the urethral opening occurs along the ventral side of the penis or on the scrotum (Baskin 2004). Defects of the male reproductive system, including hypospadias, have been produced experimentally in animal models by prenatal and perinatal administration of pesticides with estrogenic and/or antiandrogenic properties (Gray et al. 2001; Hotchkiss et al. 2004; Ostby et al. 1999a, 1999c; Wolf et al. 1999). A dose–response relationship between perinatal administration of the fungicide procymidone, an androgen receptor antagonist, and hypospadias among male offspring was noted in rats (Ostby et al. 1999b). Perinatal exposure to vinclozolin, another fungicide with antiandrogenic properties, also produced hypospadias in the male offspring of treated rats at relatively low doses (Ostby et al. 1999c). Epidemiologic studies of pesticide exposure and hypospadias have had inconsistent findings but have suffered from weaknesses in study design and exposure assessment (Bianca et al. 2003; Kristensen et al. 1997; Pierik et al. 2004; Wang and Wang 2004; Weidner et al. 1999). Only one study estimated exposure to specific pesticides or pesticide groups quantitatively. Maternal serum *p,p*′-dichlorodiphenyldichloroethylene (DDE) levels were not significantly associated with hypospadias in a nested case–control study in the San Francisco Bay area, California (Bhatia et al. 2005).

Residential proximity to an agricultural field has been used as a surrogate for pesticide exposure in epidemiologic studies of low birth weight (Xiang et al. 2000), fetal death (Bell et al. 2001), and childhood cancers (Reynolds et al. 2002). Several studies have established the usefulness of crop maps and geographic information systems (GIS) in assessing environmental exposures to agricultural pesticides (Green Brody et al. 2002; Royster et al. 2002; Ward et al. 2000). Reynolds et al. (2002) observed that childhood leukemia was significantly associated with maternal residence in census block groups with the highest use of the insecticide propargite. Distance from a treated field has been inversely correlated with pesticide levels in household dust and urinary metabolite levels in children (Loewenherz et al. 1997; Lu et al. 2000; Simcox et al. 1995).

We present results from a case–control study of hypospadias and agricultural pesticide use in eastern Arkansas using a GIS-based approach to estimate pesticide use and exposure based on maternal residential proximity to agricultural crop production.

## Materials and Methods

### Subject selection

Subjects were chosen from a population identified from an investigation of urogenital birth defects and exposure to water disinfection byproducts in Arkansas (Luben TJ, unpublished data). We identified cases of hypospadias through the Arkansas Reproductive Health Monitoring System (ARHMS), a population-based birth defect registry that uses active surveillance. Hypospadias cases were coded using the 6-digit modified British Pediatric Association/Centers for Disease Control and Prevention birth defects codes (National Birth Defect Prevention Network 2004). Eligible cases were male children diagnosed with all degrees of hypospadias born in the study area between 1998 and 2002 whose mothers resided at a geocodable address at the date of birth. ARMHS collected case information through medical record abstraction and interviews. Controls were obtained from birth certificates through the Arkansas Department of Health Vital Records Department (ADHVRD) by selecting the next two males born after each case with no congenital malformation identified on the birth certificate and frequency matched on maternal race.

**Figure 1:**
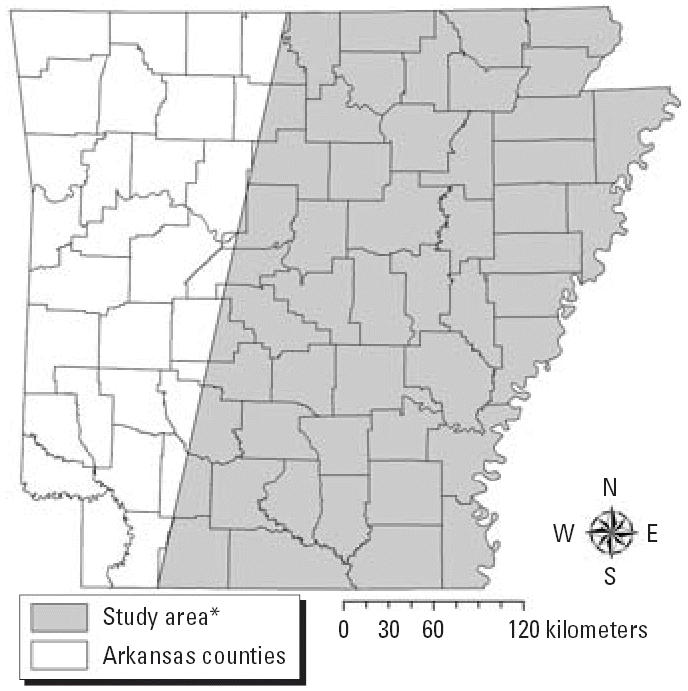
Geographic boundary of the study area. *Landsat paths 23 and 24, rows 35, 36, and 37 over Arkansas.

The study area for the analysis of pesticide exposure was prescribed by the boundaries of Landsat satellite imagery used to derive land use information [National Agricultural Statistics Service (NASS) 2004] (Figure 1). Of the 3,709 eligible addresses for the larger study, 102 (3%) were not geocoded; 52 (1.4%) represented post office or rural route boxes, 16 (0.4%) were incomplete, and 34 (0.9%) were excluded for other reasons. Within our study area, 1,201 subjects had a geocodable address. We excluded 120 births for lack of data for gestational age or land use, resulting in a final study population of 1,081 subjects (354 cases, 727 controls). Eighty-three percent were geocoded to a point within the street segment of interest, 5% were geocoded to the closest intersection, and 12% were assigned a geocode based on their zip code’s geographic centroid. Twelve percent of addresses geocoded to the closest intersection or zip code centroid represented rural route or post office boxes. Data for potential confounders and effect modifiers were obtained from birth certificates. We determined occupational exposure to endocrine-disrupting compounds (pesticides, polychlorinated organic compounds, heavy metals, and other substances) from the birth certificate data using a job-exposure matrix (Van Tongeren et al. 2002). The Colorado State University Office of Regulatory Compliance approved the use of human subjects in this study.

### Exposure assessment

We obtained georeferenced, categorized land cover data for each year from 1997 to 2002 from the NASS (2004). The data were based on summer and early fall satellite imagery mapped at a scale of 1:100,000 with a ground resolution of 30 m^2^ (NASS 2004). Categories of land use included location of major crops (corn, cotton, rice, sorghum, soybeans, and winter wheat) grown in the study area from 1997 to 2002. These crops accounted for approximately 90% of cultivated land use (NASS 2004). Average accuracy of the images was 90% for corn, 91% for cotton, 95% for rice, 80% for sorghum, 90% for soybeans, and 95% for winter wheat (NASS, unpublished data).

We obtained annual pesticide use information for each crop from Arkansas agricultural databases that included percent of acres planted and treated, average number of applications, pounds of active ingredient per acre per application, and persistence after application based on average field dissipation half-life (Agricultural Research Service 2001; NASS 2004). Application data were limited to annual statewide summaries and included only the most commonly used pesticides. We identified 116 pesticides that had been used on these crops during the study period (NASS 2004) but restricted our analyses to 38 pesticides with toxicologic evidence of reproductive, developmental, or endocrine-disrupting effects (Table 1). Applications of pesticides with strong antiandrogenic and estrogenic properties, such as vinclozolin, endosulfan, and linuron (Gray et al. 2001), were not recorded within the study area. To estimate timing of pesticide application, we established phenologic patterns specific to our study geography for each crop using weekly data from the Arkansas Agricultural Statistics Service (2005, unpublished data). We linked the phenology data to usage guidelines published by the University of Arkansas Cooperative Extension Service and the Southern Integrated Pest Management Center (Espinoza and Kelley 2004; Espinoza and Ross 2004; Slaton 2004; Southern IPM 2004a, 2004b; Soybean Commodity Committee 2004; University of Arkansas Cooperative Extension Service 2005) to estimate pesticide applications on a weekly basis. Postemergent herbicide, insecticide, and fungicide applications were included only when mention of treatment was made in weekly descriptive summaries for the year of interest. Some pesticides were applied more than once during a growing season (NASS 2004). We divided the number of months with noted applications by the average number of applications reported by NASS for the corresponding year to produce an application interval.

The exposure period was defined by a 70-day window from gestational weeks 6–16, which encompasses the critical period of *in vivo* development of male external genitalia (Baskin 2004; Yamada et al. 2003). The amount of pesticide (in pounds) applied or persisting within 500 m of each maternal residence during the critical period was used to estimate exposure (Ward et al. 2000). We used ArcGIS (ESRI, Redlands, CA) software to construct a 500-m buffer around each home and to determine the number of acres of each crop cultivated within the buffer. We linked estimated dates of crop-specific pesticide applications and their field dissipation half-lives with dates containing any portion of the exposure period for each subject. We cross-referenced pesticide use data for each application with acres grown for each crop type and calculated an estimated use (pounds of active ingredient) for each pesticide during the exposure period for each subject. The exposure metric was weighted by the probability a crop was treated with each pesticide using the percent of acres treated and planted from the statewide summaries. Exposure metrics were calculated for total pesticide use, pesticide use categorized by biologic mode of action, and pesticide use by target hormone, gland, or system subcategory as used by others (Bell et al. 2001; Reynolds et al. 2002, 2004) (Table 1). We also calculated an exposure metric based on the total and individual acres of study crops cultivated within the 500-m buffer.

### Statistical analysis

We used multivariate unconditional logistic regression to calculate risk estimates for hypospadias. Statistical models were developed and tested using SAS software (SAS Institute Inc., Cary, NC). The main effects model included maternal age, maternal race, paternal education, gestational age at birth, maternal smoking during pregnancy, and weight gain during pregnancy. We used backward elimination to identify additional potential confounders. Variables found to be associated with hypospadias (*p* < 0.05) (month of pregnancy in which prenatal care began, number of previous births, and the exposure metric representing total pesticide use) were added to the final model as well as statistically significant (*p* < 0.05) first-order interaction terms. We used the nonparametric Wilcoxon rank-sum test to compare distributions of pesticide exposures between cases and controls.

We tested categories of exposure by mechanism of action in separate models using the same set of covariates. Risk estimates were calculated by considering the exposure metrics as continuous as well as categorical variables based on observed cut points in the data. We determined the cut points for each exposure category using the Jenks optimization method in ArcGIS. The Jenks method minimized the squared deviations of the class means and set boundaries where relatively large spaces between exposure metric values occurred.

We tested five sources of potential selection bias or exposure misclassification in sensitivity analyses to determine whether alternative methods of subject and exposure classification changed risk estimates by greater than an *a priori* criterion of 10%: *a*) Mosley et al. (2002) reported lower birth defects case ascertainment than expected in seven counties located at the eastern edge of the study area. We deleted subjects from these counties, reducing the sample size to 301 cases and 600 controls. *b*) Rural residences may have greater exposure potential than urban ones (Ward et al. 2000). We identified and removed subjects (210 cases, 419 controls) with residences within census-designated urban areas. *c*) Geocoding errors could have introduced exposure misclassification (Hurley et al. 2003; Ward et al. 2005). We removed subjects (47 cases, 137 controls) whose residences were geocoded to a precision below the point within the street segment of interest. *d*) Different degrees of severity of hypospadias may have different pathogeneses (Carmichael et al. 2003). We ran the model with cases specified as primary (216) then with secondary and tertiary cases together (62). *e*) No cropland data were available for 41 cases and 66 controls because of limitations in satellite imagery. These subjects were excluded from our analyses. We compared covariate information between the 1,081 included and 107 excluded subjects using *t*-tests.

## Results

Characteristics of subjects and their parents are presented in Table 2. Most case and control mothers were Caucasian. Mean maternal and paternal ages, gestational age at birth, and birth weight were similar between case and controls groups (*p* > 0.05, *t*-test). Primiparity was slightly more common among case than control mothers, as were complications during labor or delivery (*p* < 0.05 for both). Reported alcohol use during pregnancy did not differ between groups (1% in both); however, fewer case mothers smoked during pregnancy than control mothers. This difference was not statistically significant (*p* > 0.05).

Several characteristics were significantly associated with hypospadias. Delaying pre-natal care until the third trimester resulted in a 4-fold increase in risk [odds ratio (OR)_adjusted_ = 4.04; 95% confidence interval (CI), 1.46–11.23). Older gestational age at birth and higher parity both resulted in a reduction of risk (OR_adjusted_ = 0.91; 95% CI, 0.83–0.99, and OR_adjusted_ = 0.79; 95% CI, 0.65–0.95, respectively). No other individual characteristics were significantly associated with hypospadias.

The distribution of estimated exposure to pesticides during the critical window is shown in Table 3. The analysis was limited to pesticides with potential developmental, antiandrogenic, estrogenic, or reproductive effects since they have biologically plausible modes of action to affect male urogenital development. On average, controls had higher use or persistence of study pesticides within 500 m of maternal residence, although this difference was not statistically significant (*p* = 0.22, Wilcoxon rank-sum test). Most cases (79%) and controls (78%) had at least one study pesticide applied or persisting in soil within 500 m of their residences during their respective exposure periods, but fewer (29% of cases, 33% of controls) had applications of pesticides with antiandrogenic, estrogenic, or reproductive effects. Mean exposure to bromoxynil, dicamba, prometryn, and quizalofop-ethyl was significantly lower for cases than for controls (*p* < 0.05). Means for other categories of pesticides were not significantly different between cases and controls. Among pesticides with antiandrogenic effects, only atrazine was estimated to have > 0.1 lb applied within 500 m of subject residences.

Estimated exposure to all pesticides per half-pound increase within a 500-m radius of maternal residence was negatively associated with hypospadias (OR_adjusted_ = 0.82; 95% CI, 0.70–0.96). However, when individual pesticides were aggregated by classes representing antiandrogenic, estrogenic, developmental, or reproductive effects, we found no significant associations with hypospadias (Table 4). Antiestrogenic, luteinizing hormone-disrupting, and thyroid hormone-disrupting subcategories were not significantly associated with increased risk of hypospadias (data not shown). Crop cultivation, either in aggregate or by type, within 500 m of maternal residences was also not associated with hypospadias (data not shown).

Diclofop-methyl, a herbicide applied to wheat in the study area, was significantly associated with hypospadias (Table 5). Risk of hypospadias increased by 8% for every 0.05-lb increase in the exposure metric representing diclofop-methyl use (OR = 1.08; 95% CI, 1.01–1.15). Women in the highest category of estimated exposure to diclofop-methyl (≥ 0.3 lb) had more than twice the risk of having a son with hypospadias than did unexposed mothers (OR = 2.33; CI, 1.02–5.31). Alachlor, dicamba, and permethrin were associated with a decreased risk of hypospadias. No other individual pesticides with potential developmental, antiandrogenic, estrogenic, or reproductive effects were significantly associated with hypospadias. None of the individual pesticides from other toxicologic subgroups were significantly associated with an increased risk of hypospadias (data not shown).

We evaluated the effects of yearly changes in satellite coverage, inclusion of urban residents, birth defects reporting patterns, level of geocoding, and severity of hypospadias to determine the potential for selection bias and exposure misclassification. Risk estimates were virtually unchanged in all uncertainty analyses and were not statistically significant (results not shown). Therefore, all subjects were retained in the final analyses.

## Discussion

Little prior human epidemiologic research has explored the association between exposure to pesticides and development of hypospadias. No association between exposure to agricultural pesticides by farmers and development of hypospadias in their offspring was found in a study of Norwegian farmers (Kristensen et al.1997). Weidner et al. (1999) reported nonsignificant associations between hypospadias and parental occupation in farming or gardening. A nonsignificant negative association between questionnaire-based self-reported exposure to pesticides and hypospadias was observed in a case–control study in Rotterdam, the Netherlands (Pierik et al. 2004). Paternal occupational pesticide exposure was significantly associated with hypospadias in a hospital-based case–control study in China (Wang and Wang 2004). Previous studies have not explored associations with pesticides categorized according to their specific reproductive and endocrine disrupting modes of action.

Diclofop-methyl was significantly associated with hypospadias. We found no reference to an association between diclofop-methyl and hypospadias in previous publications. Diclofop-methyl is classified as a developmental toxicant [U.S. Environmental Protection Agency (EPA) 2004]. End points in rodent studies include skeletal effects, decreased fetal weight, and distended ureters (U.S. EPA 2000). Because exposure to diclofop-methyl induces a urogenital defect in rodents, the association reported here has biologic plausibility. Delineating the mechanism of action for potentially deleterious chemicals may require long periods of study; therefore, epidemiologic evidence for an adverse effect should be followed up with environmental risk characterization (Lyons 2005). In this context, further investigation of the potential effects of diclofop-methyl on urogenital development is warranted.

Several estimated pesticide exposures showed evidence of a protective association with hypospadias. In the absence of biologic plausibility for these negative associations, the findings may have been attributed to chance. Alternatively, the reduced risk estimates observed (< 1.0) may have been partly attributed to differential ascertainment of cases among counties (Mosley et al. 2002). Underascertainment of cases could bias the risk estimates away from the null if exposures in the underascertained group differed in persons included in the study. However, our sensitivity analysis did not support the hypothesis that differential case ascertainment was responsible.

We used several novel approaches for exposure assessment. Crop phenology and canopy development data were used to build pesticide application profiles. The use of these profiles made it possible to link pesticide application data recorded on a yearly scale to gestational ages calculated on a daily scale. We developed an exposure metric based on residential proximity to crop production. Failure to include timing of pesticide application when studying congenital malformations may lead to decreased specificity for exposure and biased effect estimates (Savitz et al. 2002a). We used a critical window of gestational ages 6–16 weeks to evaluate exposures that could have affected urogenital tract development. Last menstrual period, ultrasonography, and clinical assessments were used to calculate gestational age at birth recorded in the data provided by ARHMS and ADHVRD. Gestational age can be overestimated by up to a week, depending on the method used (Barr and Pecci 2004; Savitz et al. 2002b); however, the window applied was wide enough to compensate for an error of 7 days.

Our study had several limitations. Incidence of hypospadias varies among races (Baskin 2004). The study population was relatively homogeneous with respect to race, which limited our ability to detect race-specific effects. We lacked data for several factors that might play a role in the development of hypospadias. Genetic susceptibility, familial history, use of assisted fertility technology, paternal semen quality, and exposure to other endocrine-disrupting chemicals were not included, potentially leading to residual confounding and biased risk estimates. We could not estimate home use of some pesticides [2,4-dichlorophenoxyacetic acid (2,4-D), carbaryl, chlorpyrifos] that were widely available for lawn care or indoor insecticide use. Controls were selected from among birth certificate records that did not include mention of a birth defect. Failure to include birth defect data on the certificate would have resulted in some controls being misclassified and could have biased the findings toward the null if pesticide exposures are associated with risk of the unidentified defects.

Geocoded locations were used as a surrogate for subject address data. We assumed the residential address at the date of delivery was representative of the exposure potential during the critical window of development. Residential relocation may be more common among those who have recently married or given birth (Khoury et al. 1988). Approximately 9.5% of households moved per year between 1995 and 2000 in the study area (U.S. Census Bureau 2000), suggesting that residential mobility may have introduced a relatively small amount of exposure misclassification. Inclusion was limited to subjects with a geocodable address at the date of delivery within the study area. Rural addresses may be more difficult to accurately geocode than urban ones (Ward et al. 2005) and have higher exposure potential, but only approximately 1% of the addresses not geocoded in the parent study represented rural addresses.

Limitations in our exposure assessment methods may have produced nondifferential exposure misclassification and biased our risk estimates, most likely toward the null. We assumed that our exposure metric captured exposure through the dermal and inhalation routes; however, wind conditions, ingestion, and maternal location during pesticide application contribute to exposure (Bell et al. 2001) and were not accounted for. The exposure metric was based on annual statewide summaries of pesticide application data. We were unable to account for regional differences in pesticide applications because of the limits of resolution of NASS data. Further, the cropland data layers varied in their accuracy from year to year and crop to crop (NASS, unpublished data). However, use of the cropland data layers provided a means for estimating pesticide use on a scale smaller than state or county boundaries.

Maternal exposures in our study may have been below the threshold dose required for development of hypospadias. Applications of pesticides with strong antiandrogenic and estrogenic properties were not recorded within the study area. We were limited by our ability to include only primary pesticides applied to the major crops of Arkansas. We lacked information for many pesticides that have not been tested for endocrine system function (Benbrook 1996). In addition, environmental exposure to agricultural pesticides may cause transgenerational effects not apparent until the next generation (Anway et al. 2005; Klip et al. 2002).

In summary, we applied a geographic-based exposure metric that estimated pesticide exposure during a critical window of fetal development in an epidemiologic study of hypospadias. We evaluated individual chemicals and categories grouped by biologic mode of action during the period of development relevant to hypospadias formation. The exposure assessment method reported here has potential for application in other epidemiologic studies of reproductive disorders with time-dependent features.

## Figures and Tables

**Table 1:** Pesticides applied in the study area, 1997–2002, by toxicologic group.^a^

Group/pesticide	Type	Reference
Developmental toxicants		
Bifenthrin^b^	Insecticide	U.S. EPA 2004
Bromoxynil^b^,^c^,^d^	Herbicide	U.S. EPA 2004
Dicamba^c^,^d^	Herbicide	U.S. EPA 2004
Diclofop-methyl^e^	Herbicide	U.S. EPA 2004
Diuron^b^	Herbicide	U.S. EPA 2004
Fenoxaprop^b^,^f^	Herbicide	U.S. EPA 2004
Prometryn^b^	Herbicide	U.S. EPA 2004
Propiconazole^e^,^f^	Fungicide	U.S. EPA 2004
Quizalofop-ethyl^b^	Herbicide	U.S. EPA 2004
Endocrine disruptors		
Antiandrogenic		
Atrazine^c^,^d^	Herbicide	Sanderson et al. 2002
Diflubenzuron^f^	Insecticide	Crisp et al. 1997
Iprodione^b^	Fungicide	Crisp et al. 1997
Antiestrogenic		
Atrazine^c^,^d^	Herbicide	Tran et al. 1996
Carbaryl^f^	Insecticide	Klotz et al. 1997
Cyanazine^b^,^c^	Herbicide	Tran et al. 1996
Simazine^c^	Herbicide	Tran et al. 1996
Estrogenic		
Alachlor^c^	Herbicide	Klotz et al. 1996
Atrazine^c^,^d^	Herbicide	Crain et al. 2000
Carbaryl^f^	Insecticide	Klotz et al. 1997
Permethrin^c^	Insecticide	Go et al. 1999
Trifluralin^b^,^g^	Herbicide	Rawlings et al. 1998
Luteinizing hormone disruptors		
2,4-D^b^,^c^,^d^^e^,^f^	Herbicide	Garry et al. 2001
Atrazine^c^,^d^	Herbicide	McMullin et al. 2004
Trifluralin^b^,^g^	Herbicide	Rawlings et al. 1998
Thyroid hormone disruptors		
Acetochlor^c^	Herbicide	Hurley et al. 1998
Alachlor^c^	Herbicide	Wilson et al. 1996
Aldicarb^b^	Insecticide	Porter et al. 1993
Chlorpyrifos^b^,^c^	Insecticide	Rawlings et al. 1998
Fipronil^f^	Insecticide	Hurley et al. 1998
Malathion^b^,^f^	Insecticide	Akhtar et al. 1996
Metribuzin^c^,^g^	Herbicide	Porter et al. 1993
Pentachloronitrobenzene^b^	Fungicide	Hurley et al. 1998
Pendimethalin^b^,^f^,^g^	Herbicide	Hurley et al. 1998
Trifluralin^b^,^g^	Herbicide	Rawlings et al. 1998
Not classified		
Cypermethrin^b^	Insecticide	Keith 1997
Deltamethrin^b^	Insecticide	Whaley et al. 2001
Dimethoate^b^	Insecticide	Whaley et al. 2001
Esfenvalerate^b^	Insecticide	Whaley et al. 2001
Methyl parathion^b^,^f^	Insecticide	PAN 2004
Metolachlor^b^,^d^,^g^	Herbicide	Keith 1997
Molinate^f^	Herbicide	Whaley et al. 2001
(s)-metolachlor^c^,^g^	Herbicide	Keith 1997
Zeta-cypermethrin^b^,^f^	Insecticide	Keith 1997
Reproductive toxicants		
Carboxin^f^	Fungicide	U.S. EPA 2004
Fenoxaprop^b^,^f^	Herbicide	U.S. EPA 2004
Prometryn^b^	Herbicide	U.S. EPA 2004
Quizalofop-ethyl^b^	Herbicide	U.S. EPA 2004

PAN, Pesticide Action Network.

a Some pesticides fell into more than one group.

b Applied to cotton in the study area.

c Applied to corn in the study area.

d Applied to sorghum in the study area.

e Applied to winter wheat in the study area.

f Applied to rice in the study area.

g Applied to soybeans in the study area (NASS 2004).

**Table 2:** Characteristics of subjects and their parents by diagnosis of hypospadias.

Characteristic	Cases (*n* = 354)	Controls (*n* = 727)
Subject		
Birth weight [g (mean ± SD)]	3,183 ± 700.1	3,207 ± 761.0
Gestational age [weeks (mean ± SD)]	38.2 ± 2.7	38.5 ± 2.2
1-Min Apgar score (mean ± SD)^a^	7.5 ± 1.8	8.0 ± 1.2
5-Min Apgar score (mean ± SD)^a^	8.7 ± 1.1	8.9 ± 0.8
Maternal		
Age [years (mean ± SD)]	25.2 ± 6.0	24.7 ± 5.7
White race [no. (%)]^a^	287 (81)	530 (73)
Black and other races [no. (%)]	67 (19)	197 (27)
Hispanic ethnicity [no. (%)]	7 (2)	18 (2)
Highest level of education completed [no. (%)]		
< 12th grade	50 (14)	145 (20)
High school graduate	143 (40)	306 (42)
Some college–undergraduate degree	66 (19)	153 (21)
Graduate education	63 (18)	118 (16)
Weight gain during pregnancy ± SD [lb (mean ± SD)]	32.2 ± 15.8	31.3 ± 13.6
Month in which prenatal care began (mean ± SD)	2.7 ± 1.6	2.5 ± 1.5
No. of prenatal visits (mean ± SD)	11.1 ± 4.0	11.2 ± 4.5
Primiparous [no. (%)]^a^	164 (46)	233 (32)
Used alcohol during pregnancy [no. (%)]	5 (1)	7 (1)
Smoked during pregnancy [no. (%)]	58 (16)	135 (19)
No. of cigarettes smoked per day (mean ± SD)	2.3 ± 6.0	2.0 ± 5.4
Potential occupational exposure to endocrine-disrupting chemicals [no. (%)]^b^	10 (3)	18 (2)
Complication during labor/delivery [no. (%)]^a^	114 (32)	186 (26)
Paternal		
Age [years (mean ± SD)]	28.1 ± 6.2	28.2 ± 6.7
White race [no. (%)]	251 (71)	548 (75)
Black and other races [no. (%)]	3 (1)	13 (2)
Hispanic ethnicity [no. (%)]	3 (1)	13 (2)
Highest level of education completed [no. (%)]		
< 12th grade	27 (8)	86 (12)
High school graduate	136 (38)	252 (35)
Some college–undergraduate degree	49 (14)	106 (15)
Graduate education	50 (14)	105 (14)
Potential occupational exposure to endocrine-disrupting chemicals [no. (%)]^b^	41 (12)	62 (9)

a Test of difference in means/proportions statistically significant (alpha = 0.05).

b Dichotomized using the job-exposure matrix for potential endocrine-disrupting chemicals developed by Van Tongeren et al. (2002).

**Table 3:** Descriptive statistics for pesticides (lb) applied within 500 m of maternal residences during or persisting into the critical developmental window.

	Cases (*n* = 354)		Controls (*n* = 727)	
Exposure metric	Mean ± SD	Range	Mean ± SD	Range
Study pesticides	6.8 ± 15.9	0–128.1	8.8 ± 19.7	0–180.5
Developmental toxicants	0.2 ± 1.2	0–18.2	0.3 ± 1.9	0–36.0
Bifenthrin	0.002 ± 0.01	0–0.1	0.004 ± 0.03	0–0.5
Bromoxynil^a^	0.02 ± 0.15	0–2.1	0.03 ± 0.17	0–3.1
Dicamba^a^	0.006 ± 0.05	0–0.8	0.009 ± 0.04	0–0.5
Diclofop-methyl	0.04 ± 0.2	0–1.8	0.02 ± 0.1	0–0.7
Diuron	0.1 ± 0.6	0–8.0	0.1 ± 0.9	0–15.8
Fenoxaprop^b^	0.001 ± 0.01	0–0.1	0.002 ± 0.02	0–0.3
Prometryn^a^,^b^	0.05 ± 0.5	0–8.6	0.09 ± 0.8	0–17.1
Propiconazole	0.003 ± 0.04	0–0.7	0.009 ± 0.1	0–1.6
Quizalofop-ethyl^a^,^b^	0.0006 ± 0.01	0–0.2	0.0007 ± 0.01	0 – 0.1
Endocrine disruptors				
Antiandrogenic	1.1 ± 4.3	0–52.6	1.3 ± 5.4	0–91.6
Atrazine^c^	1.1 ± 4.3	0–52.6	1.3 ± 5.4	0–91.6
Diflubenzuron	0.0001 ± 0.001	0–0.02	0.0002 ± 0.002	0–0.03
Iprodione	0 ± 0	0	0 ± 0	0
Estrogenic	2.2 ± 5.8	0–56.8	3.0 ± 7.8	0–103.2
Alachlor	0.04 ± 0.2	0–2.5	0.05 ± 0.3	0–7.3
Carbaryl	0.0001 ± 0.001	0–0.02	0.0003 ± 0.003	0–0.06
Permethrin	0.006 ± 0.07	0–1.2	0.007 ± 0.04	0–0.6
Trifluralin	1.1 ± 3.0	0–29.3	1.6 ± 4.4	0–39.2
Reproductive effects^a^	0.05 ± 0.5	0–8.6	0.09 ± 0.8	0–17.1
Carboxin	0 ± 0	0	0 ± 0	0

a Wilcoxon rank-sum test statistically significant (α = 0.05).

b Also classified as having reproductive effects.

c Also classified as having estrogenic effects.

**Table 4:** Risk estimates^a^ [no. (%)] for hypospadias with 95% CIs: exposure metrics for pesticide subcategories.

Pesticides (lb)	Cases (*n* = 354)	Controls (*n* = 727)	OR (95% CI)^a^
All study pesticides			
Per 0.5 lb applied	354 (100)	727 (100)	0.82 (0.70–0.96)
0	76 (21)	156 (21)	Referent
> 0 to < 8.2	206 (58)	397 (55)	0.94 (0.62–1.42)
≥ 8.20 to < 25.8	46 (13)	98 (13)	1.15 (0.66–2.05)
≥ 25.8	26 (7)	73 (10)	0.58 (0.28–1.20)
Developmental toxicants			
Per 0.5 lb applied	354 (100)	727 (100)	0.97 (0.87–1.07)
0	210 (59)	410 (56)	Referent
> 0 to < 0.3	120 (34)	249 (34)	0.94 (0.66–1.33)
≥ 0.3^b^	*24* (7)	68 (9)	0.76 (0.41–1.40)
Endocrine disruptors			
Antiandrogenic			
Per 0.5 lb applied	354 (100)	727 (100)	1.00 (0.97–1.03)
0	251 (71)	490 (67)	Referent
> 0 to < 2.2	67 (19)	147 (20)	0.75 (0.49–1.16)
≥ 2.2^b^	36 (10)	90 (12)	0.86 (0.51–1.44)
Estrogenic			
Per 0.5 lb applied	354 (100)	727 (100)	0.99 (0.96–1.02)
0	134 (38)	272 (37)	Referent
> 0 to < 2.7	149 (42)	282 (39)	1.11 (0.77–1.60)
≥ 2.70 to < 8.7	44 (12)	98 (13)	0.91 (0.54–1.54)
≥ 8.7	27 (8)	75 (10)	0.87 (0.46–1.64)
Reproductive toxicants			
Per 0.5 lb applied	354 (100)	727 (100)	0.89 (0.68–1.17)
0	329 (93)	647 (89)	Referent
> 0 to < 0.4	18 (5)	59 (8)	0.72 (0.34–1.53)
≥ 0.4^b^	7 (2)	21 (3)	0.46 (0.12–1.70)

a Adjusted for maternal age and race, paternal education level, weight gain during pregnancy, gestational age at birth, timing of first prenatal care visit, parity, number of cigarettes smoked per day during pregnancy.

b Third and fourth categories combined because of small cell sizes.

**Table 5:** Risk estimates^a^ [no. (%)] for hypospadias with 95% CIs: exposure metrics for individual pesticides.

Pesticides (lb)	Cases (*n* = 354)	Controls (*n* = 727)	OR (95% CI)^a^
Alachlor			
Per 0.05 lb applied	354 (100)	727 (100)	0.99 (0.96–1.03)
0	300 (85)	583 (80)	Referent
> 0^b^	54 (15)	144 (20)	0.56 (0.35–0.89)
Atrazine			
Per 0.5 lb applied	354 (100)	727 (100)	1.00 (0.98–1.01)
0	252 (71)	490 (67)	Referent
> 0 to < 3.6	71 (20)	172 (24)	0.68 (0.45–1.04)
≥ 3.6^c^	31 (9)	65 (9)	1.02 (0.58–1.79)
Bifenthrin			
Per 0.005 lb applied	354 (100)	727 (100)	0.98 (0.92–1.04)
0	308 (87)	632 (87)	Referent
> 0 to < 0.02	35 (10)	64 (9)	1.11 (0.62–1.97)
≥ 0.02^c^	11 (3)	31 (4)	0.86 (0.37–2.02)
Bromoxynil			
Per 0.005 lb applied	354 (100)	727 (100)	1.00 (0.99–1.00)
0	312 (88)	603 (83)	Referent
> 0 to < 0.1	35 (10)	98 (13)	0.79 (0.46–1.35)
≥ 0.1^c^	7 (2)	26 (4)	0.22 (0.05–1.01)
Carbaryl			
Per 0.005 lb applied	354 (100)	727 (100)	0.88 (0.59–1.32)
0	351 (99)	716 (98)	Referent
> 0^b^	3 (1)	11 (2)	0.80 (0.20–3.18)
Carboxin	0 (0)	0 (0)	—
Dicamba			
Per 0.005 lb applied	354 (100)	727 (100)	1.00 (0.98–1.02)
0	309 (87)	598 (82)	Referent
> 0 to < 0.04	34 (10)	93 (13)	0.53 (0.30–0.95)
≥ 0.04^c^	11 (3)	36 (5)	0.91 (0.38–2.14)
Diclofop-methyl			
Per 0.05 lb applied	354 (100)	727 (100)	1.08 (1.01–1.15)
0	300 (85)	630 (87)	Referent
> 0 to < 0.1	20 (6)	42 (6)	1.07 (0.54–2.15)
≥ 0.1 to < 0.3	18 (5)	39 (5)	0.78 (0.38–1.61)
≥ 0.3	16 (4)	16 (2)	2.33 (1.02–5.31)
Diflubenzuron			
Per 0.005 lb applied	354 (100)	727 (100)	0.81 (0.43–1.56)
0	351 (99)	716 (98)	Referent
> 0^b^	3 (1)	11 (2)	0.80 (0.20–3.18)
Diuron			
Per 0.5 applied	354 (100)	727 (100)	0.96 (0.85–1.07)
0	337 (95)	669 (92)	Referent
> 0^b^	17 (5)	58 (8)	0.78 (0.37–1.62)
Fenoxaprop			
Per 0.01 lb applied	354 (100)	727 (100)	0.85 (0.66–1.11)
0	343 (97)	693 (95)	Referent
> 0^b^	11 (3)	34 (5)	0.50 (0.18–1.37)
Iprodione	0 (0)	0 (0)	—
Permethrin			
Per 0.1 lb applied	354 (100)	727 (100)	1.03 (0.80–1.34)
0	340 (96)	676 (93)	Referent
> 0^b^	14 (4)	51 (7)	0.37 (0.16–0.86)
Prometryn			
Per 0.5 lb applied	354 (100)	727 (100)	0.95 (0.83–1.08)
0	338 (95)	671 (92)	Referent
> 0^b^	16 (5)	56 (8)	0.76 (0.35–1.62)
Propiconazole			
Per 0.1 lb applied	354 (100)	727 (100)	0.91 (0.69–1.21)
0	324 (92)	663 (91)	Referent
> 0^b^	30 (9)	64 (9)	0.93 (0.52–1.61)
Quizalofop-ethyl			
Per 0.01 lb applied	354 (100)	727 (100)	0.77 (0.48–1.24)
0	348 (98)	698 (96)	Referent
> 0^b^	6 (2)	29 (4)	0.81 (0.28–2.40)
Trifluralin			
Per 0.5 lb applied	354 (100)	727 (100)	0.98 (0.95–1.00)
0	175 (50)	343 (47)	Referent
> 0 to < 2.6	138 (39)	268 (37)	1.07 (0.75–1.50)
≥ 2.6 to < 8.5	29 (8)	81 (11)	0.75 (0.40–1.41)
≥ 8.5	12 (3)	35 (5)	0.60 (0.23–1.56)

a Adjusted for maternal age and race, paternal education level, weight gain during pregnancy, gestational age at birth, timing of first prenatal care visit, parity, number of cigarettes smoked per day during pregnancy.

b Dichotomized because of small cell sizes.

c Third and fourth categories combined because of small cell sizes.
